# Creating a Safety Net—Nursing Staffs' Perceptions of Conducting Suicide Risk Assessments via Telephone Within Specialised Psychiatric Care

**DOI:** 10.1002/nop2.71046

**Published:** 2026-11-05

**Authors:** Rikard Wärdig, Sally Hultsjö

**Affiliations:** ^1^ Division of Nursing and Reproductive Health, Department of Health, Medicine and Caring Sciences Linköping University Linköping Sweden; ^2^ Department of Psychiatry Ryhov County Hospital Jönköping Sweden

**Keywords:** nursing staff, psychiatry, qualitative research, suicide risk assessment, telephone assessment

## Abstract

**Aim:**

To describe nursing staffs' perceptions of conducting suicide risk assessments via telephone within specialised psychiatric care.

**Design:**

Twenty‐two nursing staff working in three psychiatric care units in central Sweden were interviewed. Phenomenographic analysis was used to analyse the data.

**Results:**

Technological advances now allow for multisensory assessments. As telephone communication lacks visual cues, nurses rely on mental imagery and intuition and listen to the patient's tone of voice. Suicide risk assessment demands collaboration and experience for collective validation. Together, these elements contribute to the creation of a safety net around the individual by the nursing staff. The study highlights a troubling shift towards standardised procedures at the expense of psychiatric competence, underscoring the need to preserve clinical expertise.

**Patient or Public Contribution:**

Psychiatric nursing staff contributed to this study by sharing their experiences through interviews.

## 1 Introduction

Globally, 700,000 people die by suicide each year, making it the fourth leading cause of death among individuals aged 15–29 years worldwide (World Health Organisation [WHO] 2025). The global perspective on suicide is complicated by cultural aspects that influence how suicide is interpreted and reported. However, a common factor in what is considered a suicidal act is behaviour, as the individual is fully aware that the action is potentially lethal (Organisation for Economic Co‐operation and Development [OECD] 2024; WHO 2024). The most significant risk factor for completed suicide, regardless of gender, is previous suicide attempts (Cooper et al. 2005; WHO 2025). A clear link between mental illness and suicide has been identified (WHO 2025). Approximately half of all individuals who die by suicide have a history of self‐harm or a psychiatric diagnosis, with depression being the most common diagnosis (McMahon et al. 2022). Statistics show that most individuals who engage in a suicidal act complete suicide on the first attempt. The first suicide attempt often involves a more lethal method (Jordan and McNiel 2020). Individuals who have received psychiatric care after a suicide attempt have, on average, made four suicide attempts during their lifetime (Tsai and Klonsky 2023). This allows for preventive measures, such as identifying and offering these individuals suicide risk assessment and relevant interventions before suicide is completed (Cross et al. 2019; OECD 2021; WHO 2025). Meaningful engagement with these individuals through telephone conversations can be a valuable approach.

## 2 Background

Of all individuals who die by suicide, 80% have had contact with healthcare services within a year before the suicide, and 40% within the month prior (Cross et al. 2019). Therefore, it is crucial that healthcare providers have sufficient competence to identify and assess patients at an elevated risk of suicide (Aflague and Ferszt 2010). When conducting a suicide risk assessment, nurses need to use both clinical judgement and various assessment tools during their conversation with the patient (Runeson et al. 2017; Aflague and Ferszt 2010). The clinical assessment involves inquiring about suicidal intent, previous suicide attempts and the current situation (Runeson et al. 2017). During face‐to‐face encounters, nurses assess suicidal ideation through both verbal and non‐verbal cues, such as poor hygiene, unusual behaviour and lack of eye contact (Wolf et al. 2018). It is also important to be attentive to changes in behaviour and mood, which may manifest in the person becoming more cheerful than before, which can be contradictory to their actual life situation (Rytterström et al. 2019).

Conducting suicide risk assessments over the phone plays a central role in suicide prevention efforts by facilitating the identification of individuals with current suicidal thoughts and supporting them in seeking psychiatric care (Gould et al. 2016). The development of digital services during the COVID‐19 pandemic reflects a shift towards providing care regardless of time and place, with an emphasis on person‐centred care, where patients take responsibility for when they use digital services for various purposes (Rosenlund et al. 2023). The increased number of phone contacts within specialist psychiatric care merits the evaluation of telephone contact as a nursing intervention (Uscher‐Pines et al. 2020; Gentile et al. 2022). During telephone consultations, nurses make their assessments solely through verbal communication, as they cannot rely on clinical observation (Kaakinen et al. 2016). It is particularly important to remain attentive to suicidal signs, as seeking care or disclosing suicidal thoughts remains stigmatised (WHO 2025). In telephone consultations with patients, nurses should be attentive, listen without judgement, be supportive and maintain an empathetic approach (Nicholas et al. 2022). Conducting assessments over the phone may lead nurses to be even more meticulous and ask more follow‐up questions to ensure a comprehensive evaluation, driven by the fear of missing suicidal signs or the fear of legal consequences if the nurse is the last person the patient speaks to (Purc‐Stephenson and Thrasher 2010). Working with individuals suffering from mental illness who are suicidal places high demands on nurses, and in psychiatric care, interdisciplinary teams are considered an effective strategy to prevent relapse and manage various symptoms with the help of different professional roles (Tomizawa et al. 2017).

Previous research shows that nursing staff find it challenging to elicit descriptions of mental illness through telephone contact. This difficulty in capturing mental illness has been shown to cause concern about not having formulated the right questions for the individual, which can also lead to worries that this may have negatively impacted the assessment (Bjorkman and Salzmann‐Erikson 2018; Wärdig et al. 2022; Pérez‐Milena et al. 2023). Nurses in primary healthcare perceived a lack of specific guidelines for handling calls related to mental illness in decision‐support designs (Wärdig et al. 2022). This created uncertainty in making contact and in assessing suicidality, which has also been demonstrated in previous studies (Bjorkman and Salzmann‐Erikson 2018). To make care accessible for individuals living with suicidality, care should be facilitated through various contact methods (WHO 2025). The telephone can be considered one such contact method. The hope is that this study can contribute knowledge to the development of these conversations.

## 3 Aims and Objectives

The aim of the study was to describe nursing staffs' perceptions of conducting suicide risk assessments via telephone within specialised psychiatric care.

## 4 Methods

### 4.1 Design

A qualitative phenomenographic approach was employed, utilising semi‐structured interviews for data collection. Phenomenography is a fitting methodology for capturing the diverse ways in which a group of individuals experiences a phenomenon (Sjöström and Dahlgren 2002). This approach seeks to understand the various ways in which phenomena are perceived in the surrounding environment, and to describe individuals' perceptions of reality and the world they engage with. In phenomenography, this is referred to as a second‐order perspective, as the descriptions are based on the informants' viewpoints rather than the researchers' (Marton 1981).

### 4.2 Sampling

A strategic sampling method was employed, aiming to recruit nursing staff with experience and the ability to contribute varied perspectives to the area under study (Polit and Beck 2021). Nursing staff with at least 1 year of experience in conducting suicide risk assessments via telephone within specialised psychiatric care were recruited for the study. The nursing staff worked in various outpatient and inpatient psychiatric clinics across three regions in central and south‐eastern Sweden. Given that suicide risk assessment via telephone is performed by different nursing staff, the sample included general nurses, psychiatric nurses and assistant nurses.

### 4.3 Setting

In Sweden, all healthcare professionals, irrespective of their profession and level of care, are expected to perform suicide risk assessments (National Board of Health and Welfare 2023). These assessments should be conducted through patient conversations and the application of suicide risk assessment instruments. Telephone counselling is a growing form of care within Swedish healthcare, and in 2022, Swedish healthcare counselling received just over 3 million calls from a total population of 10.5 million (Swedish Association of Local Authorities and Regions 2024; Statistics Sweden 2022). Telephone counselling is frequently applied within specialised psychiatric care, with nearly one in five calls concerning suicide and/or self‐harm (Vakkalanka et al. 2022). The informants were employed in various specialised psychiatric care units, such as acute psychiatric care, bipolar disorder clinics and both acute and non‐acute general psychiatric outpatient clinics.

### 4.4 Data Collection

An informational letter detailing the study's purpose and methodology was emailed to the department heads in three regions for approval to conduct the study. Following approval from the department heads, unit managers at the respective units were contacted via email with information about the study. These unit managers then informed the nursing staff in their respective units and distributed an informational letter about the study's purpose. Interested nursing staff subsequently contacted the authors to participate in the study. To capture critical differences in the sample and promote spontaneous reflections, as recommended by Booth and Marton (2002), interviews were conducted both physically (10) and digitally (12) to facilitate participation from a broader geographical area. Participants were allowed to choose the location and mode of the interviews to create a comfortable and calm interview environment to the extent possible (Polit and Beck 2021). Prior to the interviews, all nursing staff signed written informed consent. Semi‐structured interviews were utilised for data collection, as this method is deemed suitable for qualitative research intended to collect specific data within a field (Polit and Beck 2021). To raise informants' awareness about their perceptions and create space for spontaneous reflections (Booth and Marton 2002), an interview guide was developed within the research team. The interview guide contained questions aimed at exploring informants' varying perceptions of conducting suicide risk assessments via telephone. Examples of questions included: ‘What perceptions do you have of conducting suicide risk assessments via telephone?’ and ‘Can you share an example of a telephone call that went particularly well or poorly? What happened, or what did not happen?’ Follow‐up questions such as ‘Can you describe how you proceeded?’ and ‘How do you reflect on your thoughts after such a call?’ were used to deepen the responses. Initially, two pilot interviews were conducted and included in the study results, as the interview guide was found to yield responses relevant to the study's purpose. In qualitative studies, the number of interviews is not arbitrarily determined but is based on generating sufficient data to illuminate patterns and categories of the phenomenon and address the study's purpose. This implies that the exact number of nurses who should be interviewed is not predetermined (Patton 2015; Polit and Beck 2021). According to phenomenography, approximately 20 interviews are considered sufficient to provide a comprehensive view of varying perceptions of a phenomenon (Booth and Marton 2002). Analysis was conducted in parallel with the interviews until it was determined that the content of the interviews was sufficient to address the study's purpose (Patton 2015). Interviews lasted 20–60 min, were recorded on a password‐protected electronic device, and were transcribed as soon as possible to accurately capture and reflect the words and experiences shared during the interview (Polit and Beck 2021; McLellan et al. 2003). The interviews were conducted from November 2023 to January 2024.

### 4.5 Data Analysis

The phenomenographic analysis adhered to the methodology outlined by Sjöström and Dahlgren (2002). Initially, the authors meticulously read the transcripts multiple times to familiarise themselves with the data. This process was followed by identifying significant responses pertinent to the research question. Extended responses were condensed to distil essential elements, and analogous responses were grouped into a preliminary classification. This preliminary classification facilitated comparisons between categories to ensure distinct separation. Each category's unique characteristics and the varying perceptions within each category were then described. In phenomenography, the categories can be seen as a structural framework in which various experiences of a phenomenon are expressed in qualitatively different ways. Finally, a comprehensive description of each category and their logical interrelations was constructed and presented in the outcome space, the primary result in phenomenography (Marton and Booth 1997). The findings in this study were related to one another in a horizontal and hierarchical way. The analysis process was iterative and dynamic, allowing for flexibility and reflexivity. During the analysis, continuous discussions were held among the authors until consensus was reached regarding the content of the categories and their interrelations. This process was intended to enhance the trustworthiness of the results (Marton and Booth 1997). To ensure credibility, quotations from the interviews are included, allowing the reader to evaluate the relevance of the categories (Sjöström and Dahlgren 2002).

### 4.6 Ethics

The study was approved by the Ethics Committee in Linköping, Sweden (Dnr 2023‐01123‐01). It adhered to the ethical standards outlined in the Declaration of Helsinki (World Medical Association 2024). Participants were informed about the purpose and conduct of the study both in writing and verbally before the interview. The researchers complied with the General Data Protection Regulation (GDPR) and informed the nurses of their right to know the personal data being stored (Troeth and Kucharczyj 2018).

## 5 Findings

Based on the phenomenographic approach's aim of capturing a diversity of perceptions (Booth and Marton 2002), informants of varying professional categories, ages, genders and experiences in psychiatry were interviewed. According to Marton and Booth (1997), there are a limited number of ways in which a phenomenon can be experienced within a group of people. A diverse group of participants is likely to encompass these variations. A total of 22 healthcare professionals participated in the study, including 15 psychiatric registered nurses, 4 licensed nurses and 3 assistant nurses. Among the interviewees, 4 were men and 18 were women. Ages ranged from 31 to 67 years (median age 43 years). Work experience in telephone counselling within psychiatric care ranged from 1.5 to 48 years (median 17.5 years). There was no attrition.

### 5.1 The Outcome Space

The outcome space illustrates how nursing staff use themselves as clinical instruments when conducting suicide risk assessments by telephone in specialised psychiatric care. The assessment process can be understood as a continuum in which nurses initially rely on their professional judgement and adapt their actions to the needs and complexity of each individual situation.

To guide their assessments, nurses draw on experience‐based knowledge, including visualising the patient, trusting their inner voice and attending closely to vocal cues. As the assessment progresses, additional resources may be mobilised when needed. These resources include support from colleagues, consultation with other professionals, continuity in the care relationship and, in more acute situations, collaboration with external services such as emergency departments or the police.

Across the continuum, nurses described their efforts as creating a ‘safety net’ around the patient. This safety net was not viewed as a single intervention but as a dynamic and ongoing process aimed at ensuring patient safety. It was constructed through the combined contributions of clinical experience and professional judgement, relational continuity with the patient, teamwork and consultation, organisational support and access to external resources when required. Thus, the safety net functioned as an overarching framework that guided nurses' actions before, during and after the suicide risk assessment. Across the categories, suicide risk assessment by telephone was understood in increasingly comprehensive ways. The categories moved from the nurse's individual interpretative work, through relational and contextual understandings of the patient, to broader organisational and collaborative forms of responsibility. In the most comprehensive understanding, suicide risk assessment was viewed as the creation of a safety net, where patient safety was understood as a collective achievement that relied upon the integration of personal, relational, professional, organisational and societal resources. Figure 1 provides an overview of the categories identified in the findings.

**FIGURE 1:**
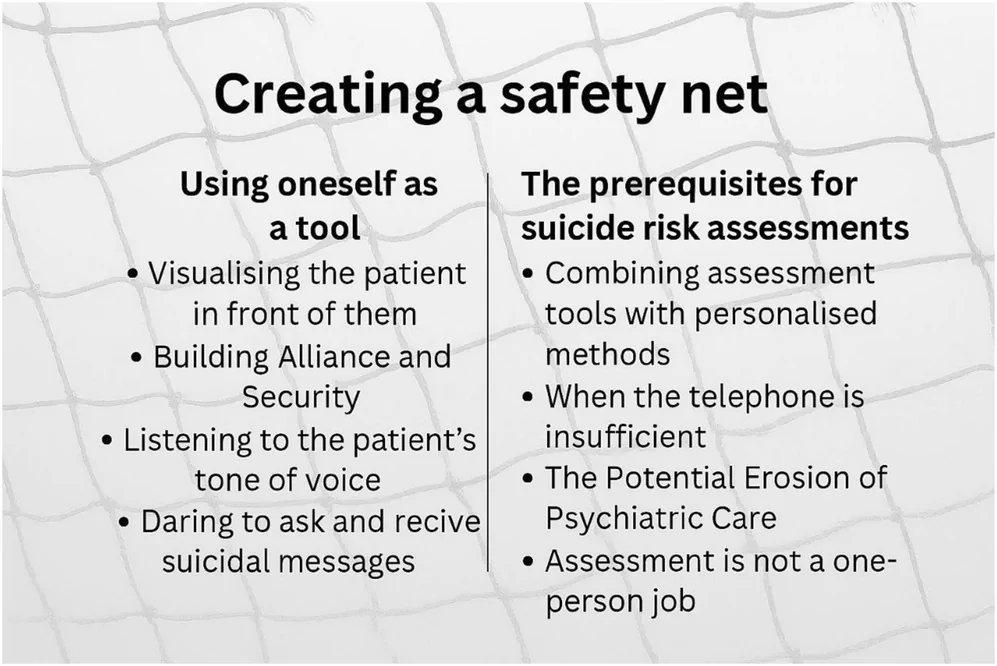
Overview of categories.

### 5.2 Using Oneself as a Tool

This category involves utilising one's senses and using oneself as a tool to create the best possible prerequisites for making an accurate assessment of the patient and their situation.

#### 5.2.1 Visualising the Patient in Front of Them

In this study, nursing staff described that when the patient was not physically present in the room, they created a sort of fictitious physical meeting by imagining the patient in front of them. Based on what the patient communicated, the nurses tried to visualise the environment the patient was in, what they were doing at the moment, and whether other people were present. Visualising the patient's environment helped to concretise the patient's needs and helped guide staff during the suicide risk assessment. Visualising the patient appeared to be more than simply imagining physical surroundings. It represented a way of creating contextual understanding in the absence of visual contact. Through this process, nurses sought to make the patient's situation more tangible and clinically accessible, despite being physically distant.

…I have heard you say that you have bought a rope and that you intend to hang yourself in the living room, and with that information, I am obligated to take action because it is the healthcare system's duty to treat and save lives.

Participants described that details conveyed during the conversation were used to construct a mental representation of the patient's circumstances. Such representations appeared to support clinical judgement by enabling nurses to interpret fragmented information as a coherent situation requiring action. Nursing staff described that telephone conversations must be allowed to take the time needed to give the patient possibilities to express their thoughts, and grant themselves the knowledge necessary to visualise the patient in front of them.

#### 5.2.2 Building Alliance and Security

The relationship and alliance were described as crucial for encouraging the patient to open up and talk about their current situation. When the nursing staff had prior knowledge of the patient, they felt more confident in the conversation with the patient. The descriptions suggest that suicide risk was not understood solely as an individual characteristic of the patient but as something that became visible within a trusting relationship. Establishing an alliance was therefore viewed as a prerequisite for gaining access to information that might otherwise remain concealed.

When it's a patient I've seen many times…I know there are suicidal thoughts, so I don't start with asking about their zest for life but rather how the suicidal thoughts are this time…It becomes more person‐centred, you could say…

Familiarity with the patient appeared to provide a frame of reference that enabled nurses to distinguish between enduring difficulties and potential changes requiring immediate attention. Nursing staff in inpatient as well as outpatient care described that it was easier to make a telephone assessment if they had observed how the patient's condition had changed over time. It also emerged that nursing staff perceived that patients were more likely to speak honestly about their status and feelings if they had previously been in contact. In telephone consultations, it emerged that the nurse does not always have prior knowledge about the patients who call. Nursing staff then had to quickly review the medical records to form an understanding of the patient in order to make their assessment. Not knowing the patient created uncertainty about whether the assessment was being conducted fairly. In this way, uncertainty was closely linked to the absence of relational knowledge. The participants described that understanding the patient over time contributed information that could not easily be obtained through structured questioning alone.

…you also do this through telephone consultations, and often you don't know the patient. You have a tight schedule, quickly go through the medical records, scroll down to some note, and form a very quick impression. Then, it becomes very stressful when you have to conduct a suicide risk assessment…

When the patient felt trust and support, staff perceived that a sense of security was created, making it easier for the patient to articulate their feelings. This led to smoother conversations with patients and the development of mutual trust.

Sometimes you just need to talk about something completely different…It could be about their pet at home or something. It creates a relationship in a different way.

#### 5.2.3 Listening to the Patient's Tone of Voice

Listening to the tone of a patient's voice involves not only perceiving the patient's tone but also one's own inner voice. The patient's tone of voice was perceived as an indicator of the patient's condition. Participants described that the meaning of what was said could not be separated from how it was communicated. Tone of voice was understood as carrying information about the patient's emotional state that extended beyond the verbal content itself. Thus, it was integrated into the suicide risk assessment. A calm tone increased concern for the patient's life, as it could be interpreted as a sign of a desire to end their lives. Therefore, calm demeanour in a patient was seen as necessitating urgent psychiatric care. Nursing staff also described that patients who expressed intense emotions and used strong words do not always intend to end their lives. Nursing staff sometimes perceive this as an expression of the patient wanting to convey and share difficult feelings. At the same time, they perceived that a low suicide risk does not diminish the seriousness of the situation and that all expressions of suicide should be taken seriously. Even if nursing staff perceived that some patients used suicide threats as a way to communicate, such expressions should never be disregarded. These descriptions indicate that suicide risk was not viewed as a straightforward reflection of emotional intensity. Rather, nurses described a need to interpret verbal and emotional expressions in relation to the broader context of the patient's situation.

The nursing staff perceived that suicide risk assessments also rely on listening to their own inner voice, developed through experience and training. This inner voice was described as a guide, particularly when staff had prior experience with patient suicides. The inner voice helped formulate relevant questions for further actions. Rather than referring to a sudden instinctive reaction, the inner voice appeared to represent an experience‐based form of knowing developed through repeated encounters with suicidal patients. It provided support when available information was limited and the assessment was characterised by uncertainty.

It's something I always carry with me, to respond to this worry or inner voice.

The inner voice was described as helping nursing staff identify concerns that could not always be articulated through formal assessment criteria. In this sense, it functioned as a complement to explicit clinical knowledge rather than a replacement for it. The inner voice could calibrate the conversation to the level where empathy and respect could be conveyed to the patient. Years of clinical work had built the ability to understand and listen to verbal and emotional messages, to truly learn to listen. However, among some nursing staff, the inner voice could lead to discomfort after difficult conversations about suicide. Thoughts and concerns about the patient could temporarily follow staff home outside the workplace, but it was perceived a priority to leave difficult feelings at work as much as possible. Leaving difficult feelings at the workplace does not indicate a cold personality, but rather reflects a professional attitude.

I can be a bit like Florence Nightingale; I sometimes want to help everyone a bit too much, but I have learned more and more not to take on their emotions.

Some nursing staff also perceived their inner voice as unreliable or not particularly useful. They described how this inner voice can conflict with the theoretical knowledge acquired during training, leading to internal conflict. Therefore, the inner voice presented a choice of whether to follow it or not. However, the limitations of telephone‐based assessment increased reliance on experience‐based judgement. In the absence of visual information, participants described drawing upon accumulated clinical knowledge to make sense of subtle cues and possible inconsistencies within the conversation.

I might have a person who says one thing, but then that person could actually be sitting with a bunch of pills in front of them or have a rope around their neck or anything, so you really have to rely on what the patient says and what you hear.

#### 5.2.4 Daring to Ask and Receive Suicidal Messages

Daring to ask patients about suicidal thoughts or plans is perceived as conveying respect towards the patient. Staying in the difficult conversation was experienced as reducing potential stigmatisation around suicidality and normalising the conversation. Nursing staff described it as important to not be intimidated when patients disclosed or confirmed thoughts and plans of suicide. There was a concern that patients might decline further dialogue if questions about suicide were posed too directly. Therefore, the nursing staff initiated conversations with a more open dialogue about life. However, this open dialogue could sometimes be misleading, as the focus tended to shift towards aspects of life other than suicidality. To ensure that suicidal thoughts were addressed, the nursing staff incorporated direct questions about suicidality into the open dialogue. However, some nursing staff perceived a certain ambiguity regarding how the conversation should be structured, as patients were considered to have individual reactions. Some could become angry or triggered by direct questions, while others described a sense of relief at being asked about, and given the chance to talk openly about, suicidality. Asking directly about suicidality was not described as being in opposition to person‐centred care. Rather, participants understood direct questions as a means of acknowledging suffering and creating opportunities for honest dialogue.

When I ask, ‘Yes, but what do you think about death? Do you really want to die, or what are your thoughts?’ I find that many say they have never been asked such a direct question before.

These descriptions suggest that direct questions could facilitate discussions about experiences that otherwise remained unspoken. Therefore, asking about suicide was understood as both an assessment strategy and a caring intervention.

The nursing staff perceived that conversations sometimes tended to stop at questions concerning suicidal thoughts and current suicidal plans. This occurred despite the understanding that suicidality also encompasses the patient's potential preparations for their survivors and life after death. Consequently, there was a lack of dialogue about what might happen next. Due to the ambiguity regarding whether questions should be asked directly, complexity was described in how suicidality should be addressed over the phone. Addressing suicidality was not only part of an assessment but also an opportunity to reach out and provide nursing care to resolve an acute situation, despite the lack of physical contact.

Nursing staff further described the importance of identifying what triggers the patient's suicidal state, even when the patient has difficulty expressing it in words. It was also considered important to listen to and understand how the patient perceived their life situation and socioeconomic conditions. Nursing staff experienced that if the patient perceived life as overwhelming, it could lead to suicidal behaviours, even though this was not expressed. Being direct via phone does not exclude a genuine conversation in which the interpersonal aspect can take place; rather, it is perceived as a foundation for good nursing care.

### 5.3 The Prerequisites for Suicide Risk Assessments

Within this category, critical supportive factors and potential pitfalls are identified with the potential of affecting the nursing staff's prerequisites for conducting suicide risk assessments.

#### 5.3.1 Combining Assessment Tools With Personalised Methods

Nursing staff described that suicide risk assessment instruments are sometimes used during telephone contacts, even though these can be perceived as ‘rigid’ or giving the conversation the ‘wrong’ focus. The assessment instruments can hinder a mutual dialogue and may therefore be completely disregarded, serve as a tool for grading suicide risk, or as an interview guide. Using the instrument as an interview guide was perceived as contributing to a good dialogue. In cases where the patient seeks care elsewhere, it can be of great value for nursing staff to easily access previous assessments in the medical record system. Based on the design of the medical record system, it could be perceived as mandatory to use these instruments, even though no one is certain that the instrument is based on a validated method. Participants did not describe standardised instruments as sufficient on their own. Instead, the instruments were understood in different ways, ranging from a structured assessment framework to a conversational support tool integrated into clinical judgement.

We use a national suicidal process model quite a lot, but when you look at it, it might not be very well‐researched, as I recall from when I checked. However, it might provide the necessary information.

The findings suggest that confidence in assessments was linked less to strict adherence to instruments and more to the nurse's ability to adapt the conversation to the individual patient's circumstances. The suicide risk assessment instrument became more of a background tool. It was there, but they used their own words when asking questions. Simultaneously, there were also perceptions that these instruments can be challenging to use. Some felt that it was not always clear when and how they should be utilised.

I don't really know, but I often start with suicidal thoughts, or ask about it somewhat loosely… Then, depending on their response, you either go deeper or move on. I don't know what else I would use.

Although nursing staff perceived that there is sometimes a place for the use of suicide risk assessment instruments, other aspects are attributed even greater value. Maintaining continuity via telephone contact and establishing an alliance with the patient are perceived as crucial factors for reliable suicide risk assessments. In these cases, simplified assessments can be conducted, in which nursing staff rely more on their own perception of the patient rather than on asking multiple questions about suicidality derived from an instrument.

The difference compared to where I work now is that we know all the patients we have, and all patients have a plan.

#### 5.3.2 When the Telephone Is Insufficient

Telephone consultations are essential, enabling broader outreach and increased efficiency. The approach became increasingly relevant during the COVID‐19 pandemic, as staff members were compelled to reach out to their patients using various digital tools. Several nursing staff in the study perceived that they consider suicide risk assessment via telephone to be a good intermediary solution when it is not possible to meet all patients in person. Participants described the telephone as simultaneously enabling and constraining suicide risk assessment. While it facilitated accessibility and continuity, the absence of visual information introduced uncertainty regarding the patient's condition.

I do not believe that proper suicide risk assessments can be conducted over the phone; that is, structured assessments. However, it is still a good complement.

Given the care level and severity of the situations at play in suicide risk assessments, they are fundamental in specialist psychiatric care. However, the presence of common psychiatric diagnoses can impede the satisfactory and safe execution of such critical assessments. Patients often exhibit intense emotions and strong expressions, making telephone‐based evaluations challenging. Particularly, patients who are emotional unstable are difficult to assess, as their suicidal tendencies are integrated into their symptoms or expressed through suicidal language.

Many, in their desperation, engage in suicide preparations and actions not because they truly wish to end their lives, but as an expression of their frustration.

Persons who are emotionally unstable often seek frequent contact with various healthcare providers, leading to inconsistencies in care and confusion about the current care plan, resulting in staff creating new plans. Psychotic symptoms also pose challenges for accurate telephone assessments, as patients may struggle to reliably communicate their condition. When psychotic symptoms dominate a patient's identity, telephone consultations are perceived as unreliable. These factors are particularly critical in suicide risk assessments, making telephone consultations less effective compared to other patient groups. Psychotic symptoms can obscure secondary suicide risk, as telephone consultations often reflect the patient's illness‐influenced worldview. These situations illustrate how participants understood the reliability of telephone assessments as dependent not only on the method itself but also on the characteristics and communicative abilities of the individual patient.

Here we observed a secondary suicide risk; he did not want to take his life, but he was so frightened in his condition that if he had continued hiding, he would have frozen to death.

Persons with symptoms of attention‐related difficulties frequently engage in anxiety‐laden calls, with patients amplifying their words through anxiety, making it challenging to discern the core issues. Despite heightened anxiety, suicide risk is rarely elevated, often requiring emotional guidance instead. Other behaviours, such as problematic substance use, also pose challenges. While telephone consultations may seem reliable, the knowledge of a patient's substance use introduces a difficult‐to‐assess suicide risk.

It is more challenging with substance use, as there is always a suicide or death risk involved in some way, making it more difficult.

Additionally, the strong influence of social media on the younger generation introduces rare suicide methods and language, complicating understanding. The generational gap can be problematic, regardless of psychiatric diagnosis. Overall, managing psychiatric patients via telephone is complex, irrespective of the reason for contact. Nursing staff perceived a more frequent use of video calls as complementary to telephone‐based suicide risk assessments by providing visual contact for more accurate evaluations.

I strongly believe in video meetings, because, frankly, there isn't much you can do over the phone beyond maintaining continuity and knowing the patient.

#### 5.3.3 The Potential Erosion of Psychiatric Care

Nursing staff perceived that psychiatric care has become increasingly standardised. There was a concern that standardised forms or interview guides may lead to the devaluation of psychiatry as a specialty. Vital parameters are frequently documented in medical records and are sometimes regarded as more important than mental status and other aspects that may be valuable in a suicide risk assessment. Participants described a tension between standardisation and professional discretion. Suicide risk assessment was understood as requiring nuanced psychiatric knowledge that could not always be captured through predefined forms and procedures.

We need to learn much, much more psychiatry again; it has disappeared.

The findings suggest that concerns about patient safety were closely connected to perceptions of diminishing opportunities to develop and maintain specialist psychiatric competence. The organisation influenced the conversations by limiting the time available in the work calendar. This also created an ethical dilemma regarding responsibility for the conversation when nursing staff felt that there had not been enough time to make a qualitative assessment. This created a feeling of inadequacy. Those working in settings characterised by high staff turnover described that they miss experienced colleagues who could serve as role models and be available in challenging situations. Furthermore, it emerged that some employers expect everyone to be able to conduct suicide risk assessments, even though their workplace does not offer an introduction.

One should know how to conduct suicide risk assessments and what it entails from the very beginning.

Nursing staff described that many experienced personnel have left, complicating the situation for the remaining staff. Staff often have to manage more patients and take on ‘more difficult cases’. This can create a sense of insecurity when it is no longer possible to seek help from experienced colleagues, which was previously routine in their own suicide risk assessments. It was considered very important that nursing staff knew each other well enough to communicate through glances or tone of voice while conducting a suicide risk assessment over the phone. In these favourable contexts, an implicit action plan was perceived among colleagues. Conversely, not being able to rely on an inexperienced colleague was seen as jeopardising patient safety.

That's kind of what I mean, that there will be two different suicide risk assessments depending on who answers…if it's a nurse with primarily somatic experience or someone with extensive psychiatric experience, there will be two different ways of handling this….

A strong desire for training in telephone‐based care was expressed. Academic knowledge in this area was often either entirely absent or acquired early in one's career, contributing to uncertainty regarding individual performance. Furthermore, the guidelines outlining the content of such conversations were perceived as vague and unclear. Questions also arose concerning responsibility in the conversation; specifically, whether staff can be held accountable for its content, or whether the organisation should address perceived gaps in staff competence and opportunity.

…they have never seen a patient with mental illness. That's how it is. They don't meet patients anymore, either; everything is phone follow‐ups at my clinic.

#### 5.3.4 Assessment Is Not a One‐Person Job

Nursing staff found it more challenging to conduct suicide risk assessments over the phone compared to in‐person meetings. Staff described that they might have made a different assessment if they had met the patient in person. They stated that they would have felt more confident in their assessment and might not have asked the patient to go to the psychiatric emergency department for further care. The phone can thus create a need for a second opinion. The assessment process was frequently described as extending beyond the individual nurse. Particularly in situations characterised by uncertainty, collaboration functioned as a means of strengthening confidence in clinical judgement.

You don't get the best assessment over the phone…There have actually been quite a few times when I've told them to go to the psychiatric emergency department just from talking to them.

Nursing staff described that in cases where the suicide risk assessment was difficult to determine, a second opinion from a colleague was needed. Consulting colleagues served both to strengthen the assessment and to provide emotional and professional support, while also ensuring that the evaluation was collectively validated. When a patient was assessed as having an elevated or high suicide risk during the phone call, all nursing staff ensured that the patient sought out the psychiatric emergency department for a medical evaluation. Nursing staff expressed that their patients often needed motivation to be willing and able to come for a second opinion. Long conversations and great commitment were sometimes required to get them to seek help. Seeking support from colleagues was not primarily described as a sign of insufficient competence. Rather, it was understood as an important strategy for achieving shared responsibility and collectively validating difficult assessments.

Of course, a lot of motivation is needed. Sometimes you actually have to say that you want them to come for an assessment because you yourself want it and because, well, you care about them. Sometimes we have to pick them up, but that's what you have to do.

Nursing staff described that suicide risk assessment via telephone occasionally required external assistance from professionals outside the organisation. This primarily involved collaboration with the police or with emergency services. This assistance was perceived as highly appreciated.

If it emerged during the telephone conversation that a patient was suicidal, nursing staff described how they enlisted the help of other colleagues to call emergency services. In such cases, the immediate response could involve keeping the patient engaged in the conversation until an ambulance arrived. If the situation was urgent, they would go directly through the police, who, unlike ambulance or on‐call doctors, could facilitate a mandatory connection to healthcare.

Then I call for my colleagues' attention to [get them to] call emergency services. I actually think the police listen to us…We trust each other, it is like that.

In its broadest sense, suicide risk assessment was therefore understood as a collective safety practice involving multiple professionals, services and organisational resources. This understanding connects closely to the overarching concept of creating a safety net around the patient.

## 6 Discussion

This study highlights that conducting suicide risk assessments via telephone in specialised psychiatric care is a dynamic and context‐sensitive process. An important contribution of this study is the conceptualisation of suicide risk assessment as the creation of a safety net around the patient. Rather than relying on a single assessment strategy, nurses described how patient safety emerged through the integration of relational, clinical, organisational and collaborative resources. Nurses initially relied on professional judgement and, depending on the complexity of the call, drew on experience‐based knowledge, including visualising the patient, trusting their intuition and paying close attention to vocal cues. As the assessment progressed, additional resources could be mobilised when needed, such as consultation with colleagues or collaboration with external services, including emergency departments, emergency medical services or the police. The safety net was therefore not a single intervention, but a dynamic process in which multiple forms of support were combined to maintain patient safety and respond to the needs of each individual situation.

An important ethical dimension emerging from the findings concerns the responsibility associated with making potentially life‐threatening assessments without visual contact. Participants described being required to evaluate suicide risk based primarily on verbal communication while simultaneously carrying a strong sense of responsibility for patient safety. This places considerable demands on professional judgement, particularly in situations characterised by uncertainty. The findings suggest that ethical responsibility in telephone‐based suicide risk assessment extends beyond the individual nurse and depends on the availability of relational, organisational and professional support structures that facilitate safe decision‐making.

Participants frequently described an ‘inner voice’ or intuitive sense as an important component of suicide risk assessment. While such intuitive impressions may help nurses identify subtle concerns that are difficult to capture through standardised procedures alone, intuition should not be regarded as inherently reliable or unproblematic (Melin‐Johansson et al. 2017). Intuition is shaped by personal and professional experience and should therefore not be regarded as inherently reliable or free from bias (Benner and Tanner 1987). This may be particularly relevant in telephone‐based assessments, where judgements rely primarily on verbal communication and where visual and contextual cues are unavailable. Consequently, intuition may be best understood as a complementary source of clinical knowledge that can guide further exploration, rather than as a substitute for structured assessment and clinical dialogue. From an ethical perspective, reliance on intuition also introduces challenges. When visual information is unavailable, nurses may be required to make critical decisions based on incomplete information. Balancing experience‐based judgement with structured assessment therefore becomes an important ethical consideration in efforts to minimise the risk of both overestimating and underestimating suicide risk.

Our results indicated that nursing staff, as part of their assessment, evaluated the patient's tone of voice to discern potential suicidality. A calm and factual demeanour leads to a sense among staff that suicidality may be present. Previous research has shown that a blunted facial expression, cautious movement patterns, lowered tone of voice and reduced speech tempo and volume have been observed in patients admitted to psychiatric clinics following suicide attempts (Galatzer‐Levy et al. 2021). All of these expressions have been identified in individuals with depression, and one explanatory model suggests that they are triggered by decreased levels of serotonin (Morrissette and Stahl 2014; El Mansari et al. 2010). A suicide risk assessment conducted via telephone is inherently limited to factors that can be discerned through listening. Other digital tools for remote communication could therefore potentially be valuable. These tools could assist nursing staff in visualising the patient by providing an actual image of the patient's environment. In our findings, nursing staff needed to create an imaginary picture; something modern technology could provide. The nurses in this study felt that the COVID‐19 pandemic facilitated the adoption of digital meetings, which can be a valuable complement to telephone consultations in psychiatric care. During the COVID‐19 pandemic, the use of digital tools increased worldwide (Venville et al. 2021). Communicating digitally was perceived as convenient and practical for some patients, while others found it stressful to see themselves on the screen during the conversation. Among oncology nurses, video calls have been shown to facilitate patient contact and follow‐up compared to physical visits (Rygg et al. 2021). An increased use of video calls for suicide risk assessments could create conditions for more reliable assessments than calls conducted via telephone, as nurses would be able to utilise more of their senses during the evaluation process.

Our results showed that nurses perceived telephone consultations to be time‐saving and to facilitate patients' access to healthcare. Previous research has demonstrated the advantages of telephone consultations over physical visits, as they increase the accessibility of healthcare services and reduce the workload of staff (Brant et al. 2016). Telephone consultations have proven to be an accessible tool for nurses and have been shown to provide high patient satisfaction (Souza‐Junior et al. 2016). It was found that patients who received telephone consultations with nurses at psychiatric clinics had fewer visits and admissions to psychiatric care. On the other hand, patients prefer physical meetings over digital communication with healthcare providers when given the choice (Hawrysz et al. 2021). This may be because not all patients perceive recurring telephone calls over time as positive, and they may find it burdensome to have prolonged and frequent contact with healthcare services (Michaud et al. 2021). In contrast, our study found that nurses perceived long‐term contact as fostering a trusting and honest care relationship. Allowing patients to be involved and to influence their care, rather than following a predetermined plan, is one of the most important foundations of psychiatric care (Swenne and Skytt 2014). When patients decide when to contact healthcare services via telephone, it enables them to take responsibility for assessing their need for help, which promotes person‐centred care (Rosenlund et al. 2023). It is important for nursing staff to be attentive to patients' varying wishes and needs, even when the patients themselves initiate contact with healthcare services. Conducting suicide risk assessments is complex, as patients do not always disclose or acknowledge suicidal thoughts or plans (Oquendo and Bernanke 2017). Therefore, it is essential for nursing staff to ask questions to identify whether the patient is suicidal (McDowell et al. 2011), and to have tools available to ask the right questions to assess the patient's suicide risk. In this study, nursing staff sometimes described difficulties in daring to ask questions due to potential patient reactions. This could lead to the conversation switching focus to topics other than suicidality, its underlying causes and what happens after a suicide.

To reduce perceived stress and feelings of uncertainty, it is important to have a functional team in the workplace and good communication among team members, where asking for support and advice is encouraged (Vinckx et al. 2018; Tomizawa et al. 2017). For nurses, it is significant to reflect with colleagues, as this confirms nurses' own assessments and highlights questions that need to be posed to the patient (Aflague and Ferszt 2010). Our results showed that this support sometimes falters.

The findings also raise ethical concerns regarding the organisational conditions under which suicide risk assessments are conducted. Participants described situations where access to experienced colleagues was limited due to staff turnover, shortages of psychiatric expertise and increasing workload. Although responsibility for suicide risk assessment is often placed on individual clinicians, the findings suggest that organisational factors strongly influence their ability to make safe and well‐founded decisions. Consequently, ethical responsibility should not be regarded solely as an individual matter but also as an organisational obligation to provide adequate competence, supervision and opportunities for collegial consultation.

Informants reported that psychiatric expertise is being depleted, as new staff are recruited from somatic care and older colleagues with extensive experience begin to retire. Previous research presents a problematic picture of the current state of psychiatry (Gabrielsson et al. 2021). Nursing students perceive the psychiatric specialty as the least attractive (Happell et al. 2015), and there is a conflict between the ideals and the reality faced by nursing staff in clinical settings (Gabrielsson et al. 2016). Psychiatry holds a low status as a specialty, and nurses are expected to know their place in a context where nursing is subordinate to medicine (Lakeman and Molloy 2018). This is highly peculiar; nursing should arguably be the most prominent element within a psychiatric context, where there are substantial opportunities to make a meaningful impact.

The results of this study highlighted that staff sometimes felt uncertain in their assessments and therefore perceived themselves to be in need of a second opinion from a doctor or colleagues at the psychiatric clinic. In such cases, external collaboration might also be necessary; for example, with the police or fire department if immediate intervention was required. The feeling of uncertainty in their assessments can be interpreted in several ways. It could be that the nursing staff are unsure about a task that falls within their responsibilities (Terry 2020), or there may be a concern about having forgotten to ask important questions during the conversation (Bjorkman and Salzmann‐Erikson 2018; Wärdig et al. 2022; Pérez‐Milena et al. 2023). Alternatively, it could be about nursing staff leveraging the strength of a team to promote patient safety (Grant and Lusk 2015), using both belt and braces in an assessment that potentially involves life and death. Seeking a second opinion may therefore also be understood as an ethical strategy aimed at sharing responsibility and reducing the risk of harm. In high‐stakes situations characterised by uncertainty, collaborative decision‐making appeared to function not only as a clinical resource but also as a means of strengthening ethical accountability towards the patient.

Nursing staff in this study highlighted that certain psychiatric issues are particularly challenging, especially those involving emotional instability or individuals who have difficulty expressing themselves verbally. According to WHO (2025), it is crucial that all suicidal statements are taken seriously, and local cultures should not develop where nursing staff begin to interpret suicidal statements as the patient being manipulative (Simpson et al. 2021). In cases where suicide risk assessments are considered challenging due to the patient's difficulties communicating, the perspectives and experiences of close relatives could be valuable. Relatives of patients who died by suicide often feel excluded from care both before and after the suicide (Hultsjö et al. 2022). This is also reflected in this study, as relatives are not mentioned in the results. The benefits of involving the patient's relatives are significant, as they can help create a sense of security by addressing the patient's suicidal thoughts (Momeni et al. 2022; Edwards et al. 2021). Relatives also possess information that is significant in suicide prevention efforts (Hultsjö et al. 2022). Their involvement could provide useful information and create a sense of security for all involved.

The nurses in this study emphasised the importance of a secure and trustworthy approach to make the patient comfortable and willing to answer questions honestly. It has been shown that it is easier for patients to acknowledge suicidal thoughts when the nurse focuses on building a relationship, shows genuine interest and demonstrates empathy for the patient during the conversation (Ganzini et al. 2013; Richards et al. 2019). Several studies describe suicide risk assessments as a demanding task where nurses may feel uncertain about their evaluations and experience a sense of responsibility for a person's life (Jansson and Graneheim 2018; Derblom et al. 2021). To achieve patient safety and enhance staff's ability to engage in open dialogue with the patient, it is crucial for nursing staff to listen to the patient—regardless of whether the assessment is conducted via telephone or in person (Jennissen et al. 2022; Svensson 2022). Often, this dialogue is more important than standardised risk assessments. Sweden is currently undertaking a broad, cross‐sectoral effort to implement new suicide prevention guidelines. The initiative aims to strengthen preventive measures at both national and local levels, with a particular focus on understanding the individual's subjective experience of suicidality. Rather than relying solely on risk factors and statistical models, the approach emphasises the importance of engaging with the individual's narrative, needs and life context. This shift calls for the development of healthcare practices—especially in communication, documentation and responsibility allocation—aligned with a more person‐centred and empathetic approach (Public Health Agency of Sweden 2025). Taken together, the findings suggest that suicide risk assessment by telephone cannot be understood solely as an individual clinical task. Rather, it represents a complex process of judgement, relationship‐building, collaboration and ethical responsibility. The concept of creating a safety net captures how patient safety is achieved through the integration of personal, relational, organisational and societal resources.

### 6.1 Strengths and Limitations of the Work

The study included registered nurses, both general and psychiatric specialists and assistant nurses. The sampling was a deliberate choice made by the authors to capture a diversity of experiences and perspectives on conducting suicide risk assessments via telephone (Booth and Marton 2002). The inclusion of participants from different professional backgrounds may be viewed as both a strength and a limitation. While it enabled the capture of diverse perspectives, differences in psychiatric knowledge and clinical responsibility may also have contributed to variations in how participants interpreted and discussed suicide risk assessment. On the other hand, this approach to sampling is well aligned with the fundamental principles of phenomenography (Booth and Marton 2002).

Interviews were conducted in locations chosen by the participants themselves to ensure a calm and secure interview environment (Polit and Beck 2021). Ten interviews were conducted in person in private rooms, while 12 were carried out via Zoom across 3 mid‐sized regions in central Sweden. In line with the phenomenographic approach, the phenomenon under investigation is considered independent of geographical context. What matters for the results is the social, spatial and temporal context in which the participant's experience is situated (Booth and Marton 2002).

‘Transferability’ refers to the extent to which the findings can be applied in other contexts (Lincoln and Guba 1985). To enable readers to assess the degree of transferability, detailed descriptions of the sampling strategy, data collection procedures, participants, workplaces and professional backgrounds have been provided (Patton 2015). A semi‐structured interview guide was developed by the research team to stimulate participants' awareness of their own thoughts and to allow for spontaneous reflection—an approach that, according to Booth and Marton (2002), facilitates access to genuine experiences. Sjöström and Dahlgren (2002) argue that phenomenography is particularly well‐suited for nursing research.

The fact that twelve interviews were conducted digitally may have influenced the interpersonal dynamics typically present in face‐to‐face interviews. However, based on the richness and quality of the data, this was not considered a limitation. Qualitative research makes the depth of interviews more valuable than their quantity. After 17 interviews, no new data emerged, suggesting data saturation and enhancing the study's credibility (Patton 2015).

The analysis was conducted by psychiatric specialist nurses with extensive clinical and research experience, which provided a deep understanding of the phenomenon but also posed the risk of interpretive bias due to preunderstanding (Sjöström and Dahlgren 2002). The potential influence of researchers' preconceptions on study outcomes is a well‐documented concern (Polit and Beck 2021). To mitigate this risk, the authors engaged in critical self‐reflection and collaborative discussions aimed at minimising the impact of their preunderstandings on the findings.

## 7 Conclusion

The study demonstrates that conducting suicide risk assessments via telephone is both challenging and complex, requiring nursing staff to possess a high level of psychiatric competence and confidence in their professional abilities. In the absence of visual cues, assessing suicide risk becomes a particularly demanding task. Nurses described drawing on experience‐based knowledge, including mentally visualising the patient, attending to vocal cues and relying on professional judgement when making assessments under conditions of uncertainty.

A central finding was that suicide risk assessment was understood as the creation of a safety net around the patient. Patient safety was not viewed as the outcome of a single assessment or intervention, but as a dynamic process involving the integration of personal, relational, organisational and collaborative resources. Patients who experience difficulties communicating their situation may also benefit from the involvement of significant others, who can contribute valuable contextual information to the assessment process. In this process, collegial consultation, continuity of care and opportunities for second opinions were perceived as essential components of patient safety and collective validation of clinical judgement.

The findings also highlight important ethical challenges associated with making potentially life‐threatening assessments without visual contact. These challenges underscore the importance of adequate organisational support, access to experienced colleagues and opportunities for shared clinical decision‐making. Although the findings are context‐specific and cannot be assumed to reflect psychiatric care more broadly, they point to a potentially problematic reliance on standardised assessment procedures that may risk narrowing the space for professional judgement and clinically grounded expertise in suicide risk assessment.

## Author Contributions

**Rikard Wärdig:** conceptualization, investigation, writing – original draft, methodology, validation, writing – review and editing, formal analysis, data curation. **Sally Hultsjö:** conceptualization, writing – original draft, investigation, methodology, validation, writing – review and editing, formal analysis.

## Funding

The authors have nothing to report.

## Conflicts of Interest

The authors declare not conflicts of interest.

## Data Availability

The data that support the findings of this study are available from the corresponding author upon reasonable request.
